# Technical Considerations for Pancreaticoduodenectomy with Preservation of the Right Gastroepiploic Vessels after Proximal Gastrectomy: A Case Report

**DOI:** 10.70352/scrj.cr.26-0538

**Published:** 2026-09-17

**Authors:** Shingo Nakashima, Yusuke Yamamoto, Kyoka Mouri, Tomohito Maeda, Masato Mitsuda, Nobuyuki Watanabe, Hitoshi Hino, Yuen Nakase, Yasushi Suganuma, Atsushi Shiozaki

**Affiliations:** 1 Department of Surgery, Nara City Hospital, Nara, Nara, Japan; 2 Division of Digestive Surgery, Department of Surgery, Kyoto Prefectural University of Medicine, Kyoto, Kyoto, Japan

**Keywords:** pancreaticoduodenectomy, proximal gastrectomy, right gastroepiploic vessels

## Abstract

**INTRODUCTION:**

Pancreaticoduodenectomy (PD) in patients with a history of proximal gastrectomy poses a significant challenge because the remnant stomach depends largely on the right gastric artery and right gastroepiploic artery (RGEA) for its blood supply. As these vessels are routinely resected during standard PD, their preservation requires alternative surgical strategies and meticulous techniques.

**CASE PRESENTATION:**

An 81-year-old man who underwent postoperative follow-up at our institution was found to have an incidental pancreatic head tumor on CT. He had previously undergone robot-assisted proximal gastrectomy for esophagogastric junction cancer. After neoadjuvant chemotherapy with gemcitabine plus S-1, PD with preservation of the RGEA and right gastroepiploic vein (RGEV) was planned. Gastric transection, which is usually conducted at an early stage, was intentionally delayed until immediately before the dissection of the gastroduodenal artery (GDA) to minimize tension on the preserved vessels. Indocyanine green fluorescence imaging confirmed adequate perfusion of the remnant stomach. Reconstruction was performed using the modified Child method. A pancreatojejunostomy was created ventral to the preserved RGEV, allowing the vein to course dorsal to the anastomosis and separate the preserved GDA–RGEA axis from the anastomosis, thereby minimizing the risk of vascular compromise in the event of a postoperative pancreatic fistula. The postoperative course was complicated by a pancreatic fistula and cholangitis, both of which were managed conservatively, and oral intake was well tolerated. Eight months after surgery, the patient remains well and is receiving adjuvant chemotherapy, with no significant postoperative weight loss and no evidence of recurrence.

**CONCLUSIONS:**

PD with preservation of the RGEA/RGEV is a feasible and effective option for patients with a history of proximal gastrectomy. Careful patient selection and technical modifications may enable safe oncological resection while preserving the remnant gastric perfusion and postoperative function.

## Abbreviations

CA19-9
: carbohydrate antigen 19-9
CEA
: carcinoembryonic antigen
GDA
: gastroduodenal artery
ICG
: indocyanine green
PD
: pancreaticoduodenectomy
RGA
: right gastric artery
RGEA
: right gastroepiploic artery
RGEV
: right gastroepiploic vein

## INTRODUCTION

PD is a standard surgical procedure for malignant tumors of the pancreatic head; however, it becomes technically challenging in patients with a history of proximal gastrectomy or esophagectomy.^1)^ With the expanding indications for and increasing use of proximal gastrectomy in recent years, surgeons are more frequently encountering patients who require PD after this procedure.^2)^ After proximal gastrectomy, the blood supply to the remnant stomach relies predominantly on the RGA and RGEA, while the RGEV plays an important role in venous drainage.^3)^

In standard PD, the RGA and right gastroepiploic vessels are routinely divided as part of the oncological resection. Consequently, PD following proximal gastrectomy often requires completion total gastrectomy to prevent ischemic complications in the remnant stomach.^4)^ However, the completion of total gastrectomy may lead to impaired postoperative nutritional status and reduced QOL, particularly in older patients.^5)^ Recent reports have demonstrated the feasibility of preserving the RGEA and RGEV during PD in selected patients, thereby allowing for preservation of the remnant stomach.^3,6–8)^ Nevertheless, these procedures require careful preoperative assessment and technical modifications to ensure oncological safety and adequate gastric perfusion. Herein, we report a case of PD with preservation of the RGEA and RGEV after proximal gastrectomy and describe the technical modifications aimed at reducing the risk of vascular complications while maintaining remnant gastric perfusion.

## CASE PRESENTATION

**Fig. 1:**
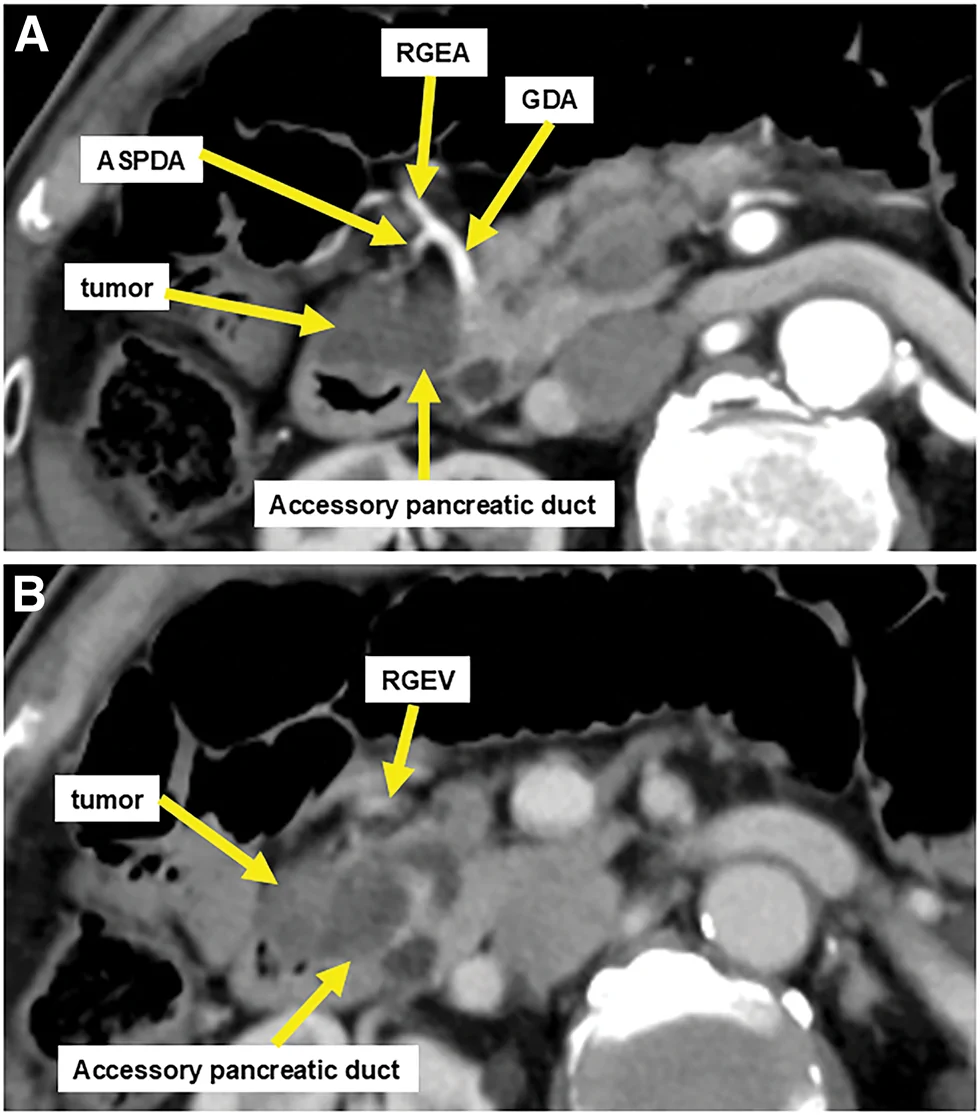
Preoperative CT findings. (**A**) CT revealed an approximately 25-mm tumor in the pancreatic head. The tumor was located approximately 11 mm from the GDA–RGEA axis. (**B**) The tumor was located approximately 13 mm from the RGEV. ASPDA, anterior superior pancreaticoduodenal artery; GDA, gastroduodenal artery; RGEA, right gastroepiploic artery; RGEV, right gastroepiploic vein

An 81-year-old man with a history of oncological surgery underwent postoperative surveillance at our institution. He had undergone robot-assisted proximal gastrectomy with esophagogastrostomy using the double-flap technique (Kamikawa method) for esophagogastric junction cancer 2 years earlier and laparoscopic anterior sectionectomy for hepatocellular carcinoma 1 year earlier. During routine follow-up CT, an approximately 25-mm mass was incidentally detected in the pancreatic head (**Fig. 1**). Endoscopic US-guided fine-needle aspiration revealed poorly differentiated adenocarcinoma. The serum tumor marker levels were elevated, with a CEA level of 30.9 ng/mL and a CA19-9 level of 248 U/mL. Based on these findings, the patient was diagnosed with resectable pancreatic head cancer without lymph node metastasis (cT2N0M0, cStage IB, according to the Union for International Cancer Control [UICC] 8th edition). According to the Global Leadership Initiative on Malnutrition (GLIM) criteria, he did not meet the criteria for malnutrition. His overall performance status and physiological condition were considered adequate for major surgery. Therefore, 2 courses of neoadjuvant chemotherapy with gemcitabine plus S-1 were administered in accordance with the Japanese Clinical Practice Guidelines for Pancreatic Cancer (2025 edition) (gemcitabine 1000 mg/m^2^ on days 1 and 8, plus S-1 100 mg daily on days 1–14 of a 21-day cycle). From day 8 of the first course, the gemcitabine dose was reduced to 800 mg/m^2^ and the same dose was used throughout the second course. Additionally, the dose of S-1 was reduced to 80 mg daily from day 1 of the second course.

Preoperative imaging demonstrated that the pancreatic tumor was located approximately 11 mm from the GDA–RGEA axis (**Fig. 1A**) and approximately 13 mm from the RGEV (**Fig. 1B**), supporting the technical and oncological feasibility of vessel preservation. The RGA was not clearly identified on preoperative imaging. If present, the RGA was planned to be ligated and divided during surgery, whereas preservation of the GDA–RGEA axis was planned because the tumor was not adjacent to these vessels. D1+ lymphadenectomy had been performed during the previous proximal gastrectomy for gastric cancer. In the present surgery for pancreatic cancer, D2 lymphadenectomy was planned. Based on these considerations, PD with preservation of the RGEA and RGEV was planned. The surgical strategy included abandoning resection if unresectable factors were encountered intraoperatively. In addition, vascular reconstruction of the RGEA was planned in the event of inadvertent injury to the vessel.

**Fig. 2:**
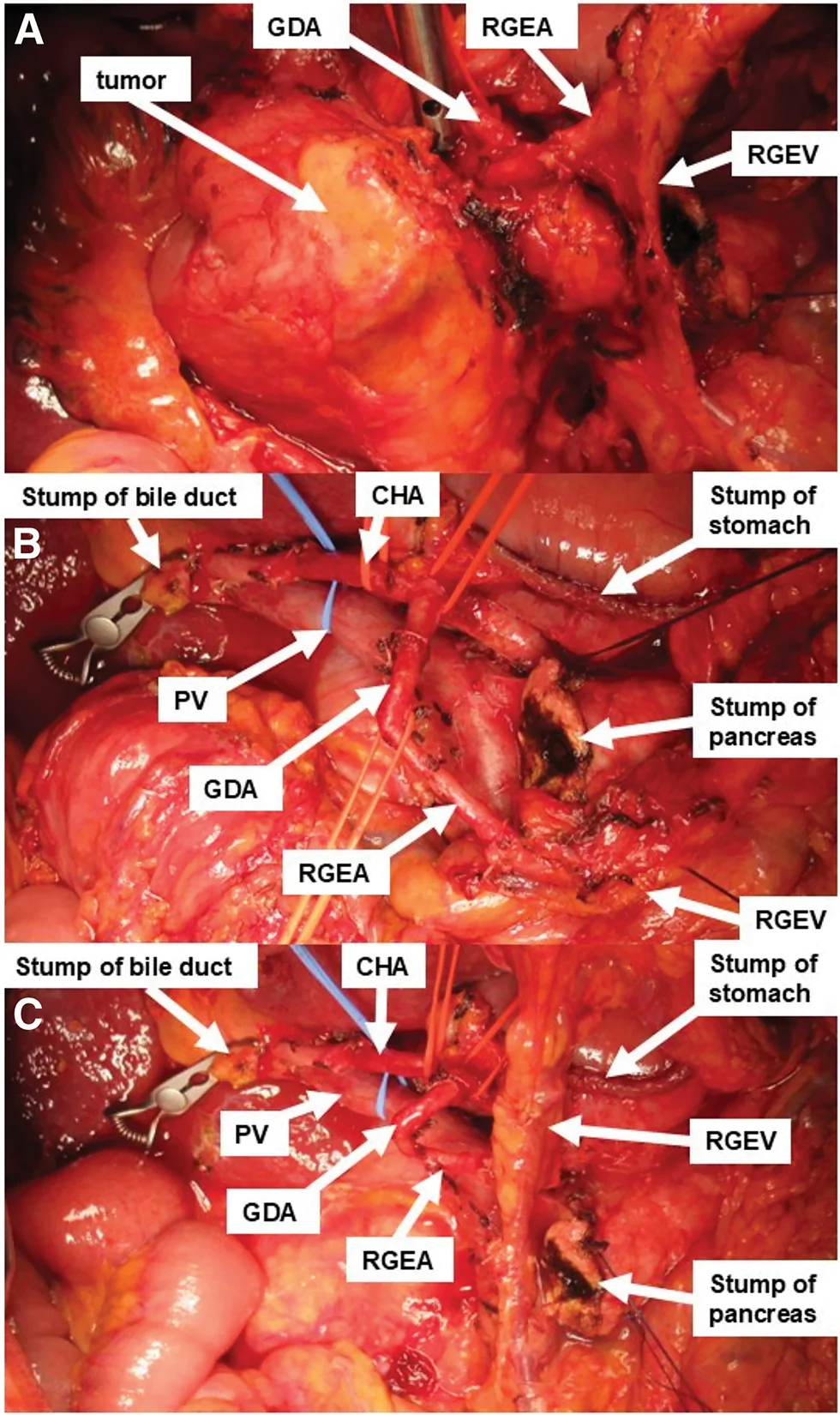
Intraoperative findings during PD. (**A**) Preservation of the GDA–RGEA axis and RGEV during pancreatic dissection. (**B**, **C**) Operative field after specimen removal demonstrating preserved vascular anatomy. CHA, common hepatic artery; GDA, gastroduodenal artery; PD, pancreaticoduodenectomy; PV, portal vein; RGEA, right gastroepiploic artery; RGEV, right gastroepiploic vein

**Fig. 3:**
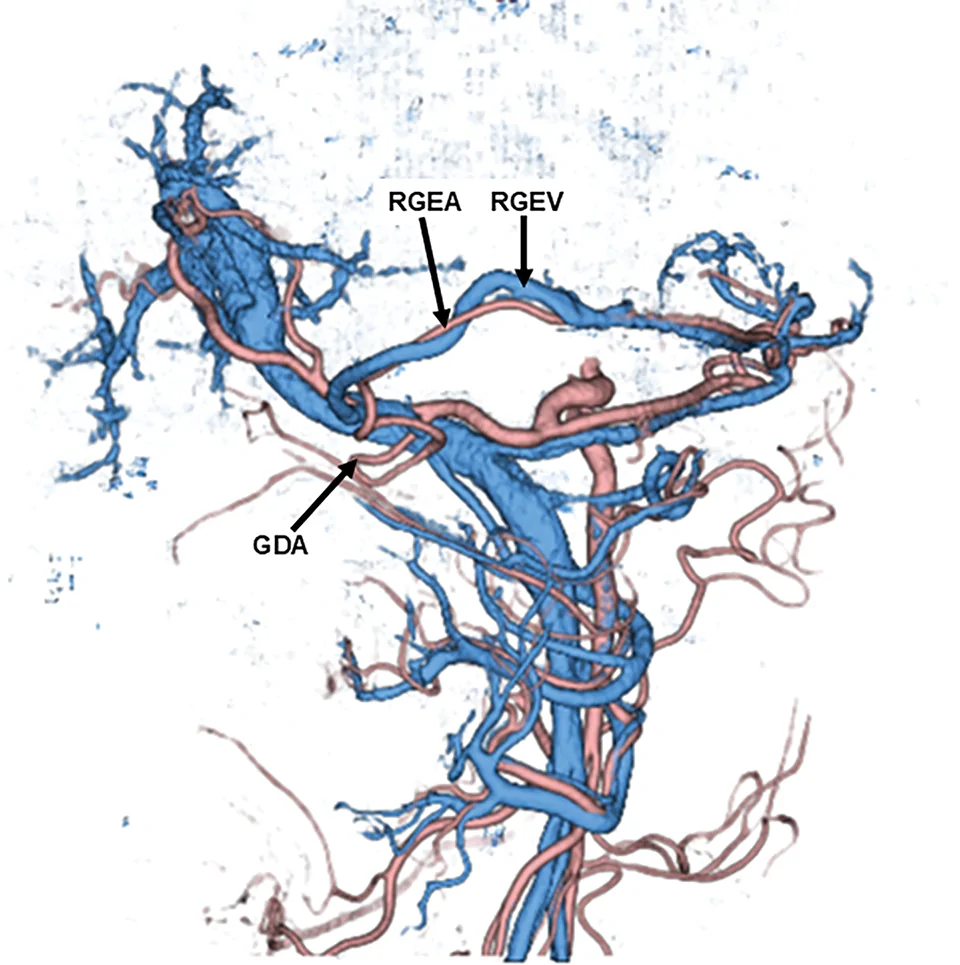
Postoperative CT findings. CT demonstrated good patency of the preserved RGEA and RGEV. GDA, gastroduodenal artery; RGEA, right gastroepiploic artery; RGEV, right gastroepiploic vein

**Fig. 4:**
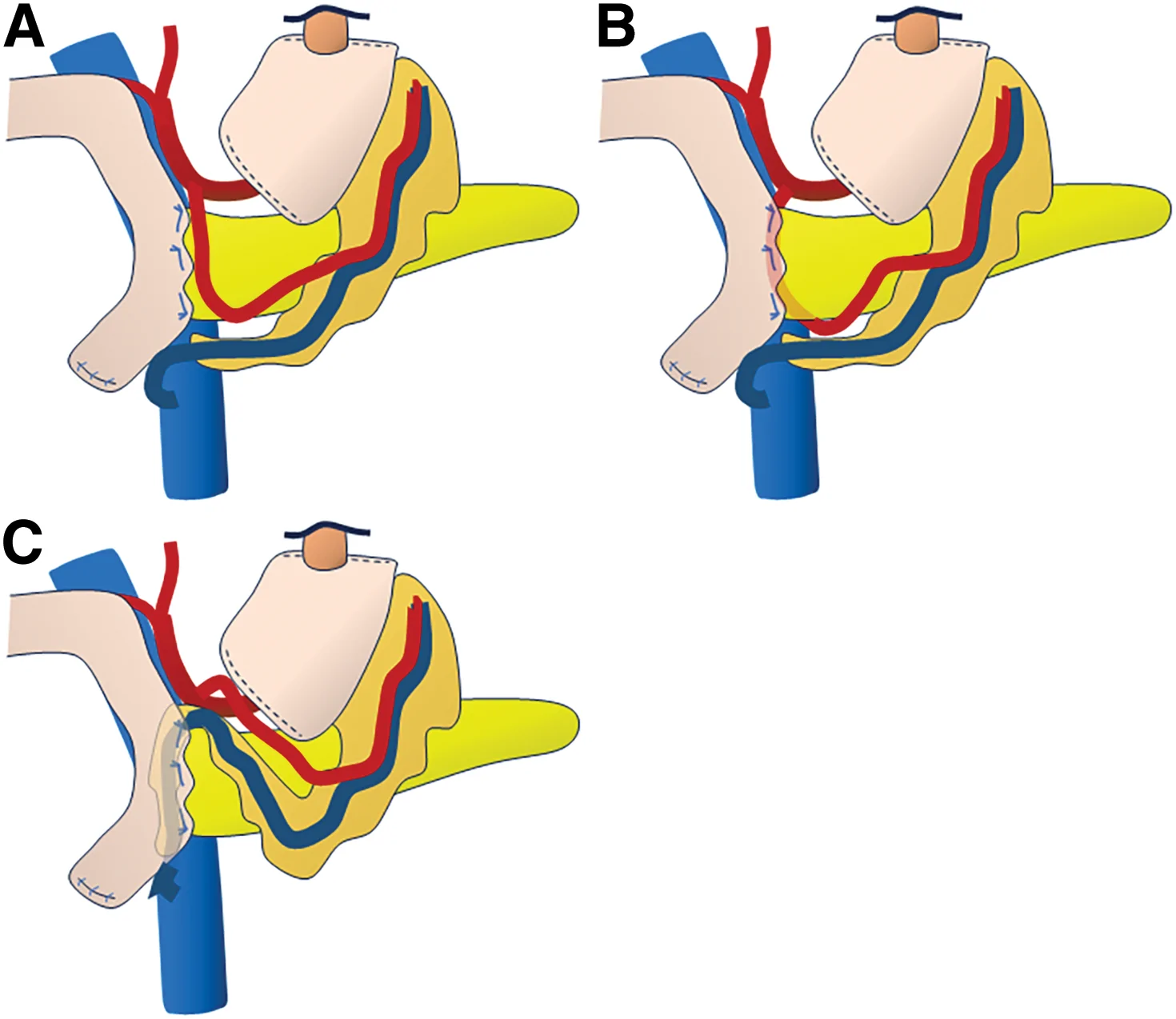
Reconstruction patterns and protection of the preserved GDA–RGEA axis. (**A**, **B**) The preserved GDA–RGEA axis remains adjacent to the pancreatojejunostomy. (**C**) Reconstruction in the present case. The pancreatojejunostomy was created ventral to the preserved RGEV, allowing the vein to course dorsal to the anastomosis and spatially separate the preserved GDA–RGEA axis from potential pancreatic leakage. GDA, gastroduodenal artery; PHA, proper hepatic artery; PV, portal vein; RGEA, right gastroepiploic artery; RGEV, right gastroepiploic vein

**Fig. 5:**
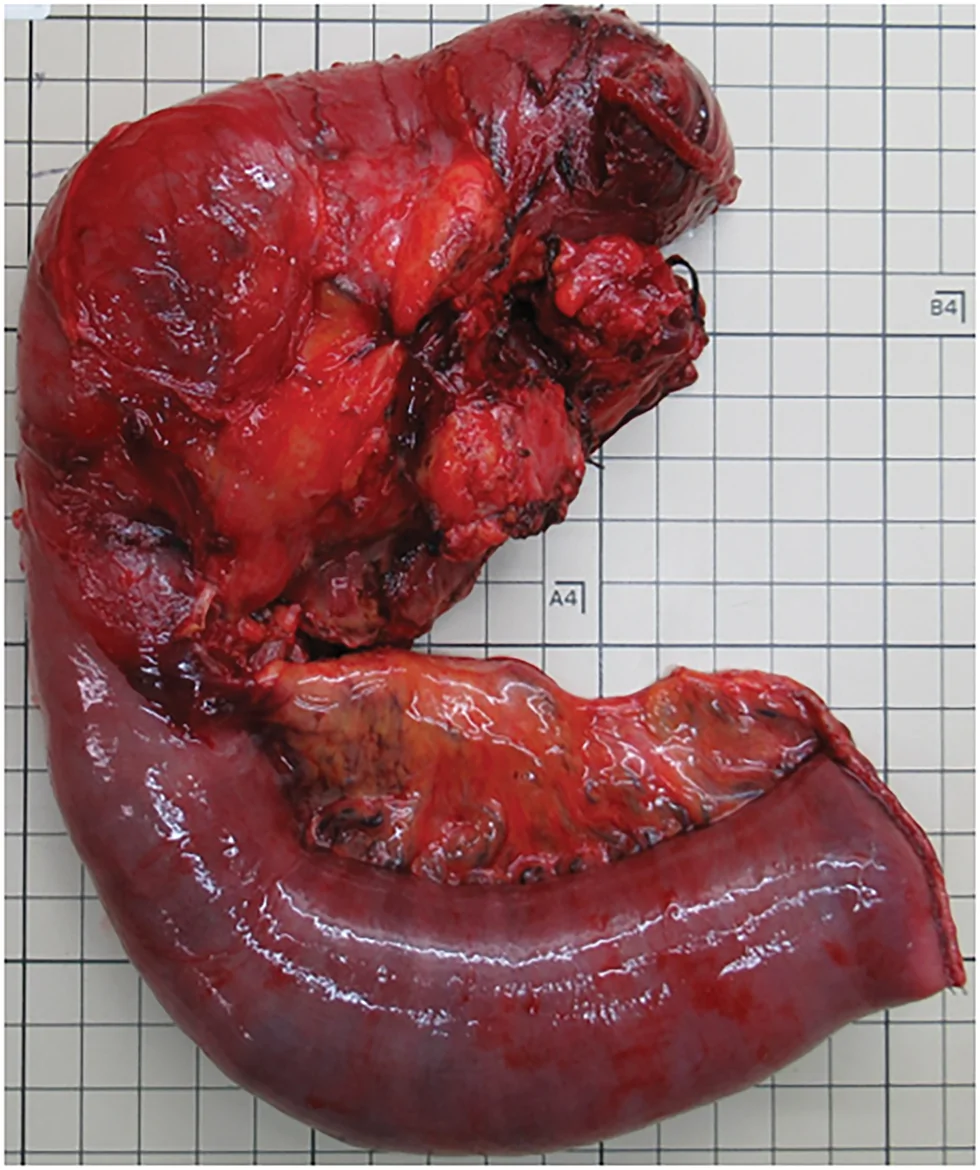
Macroscopic findings of the resected specimen.

**Fig. 6:**
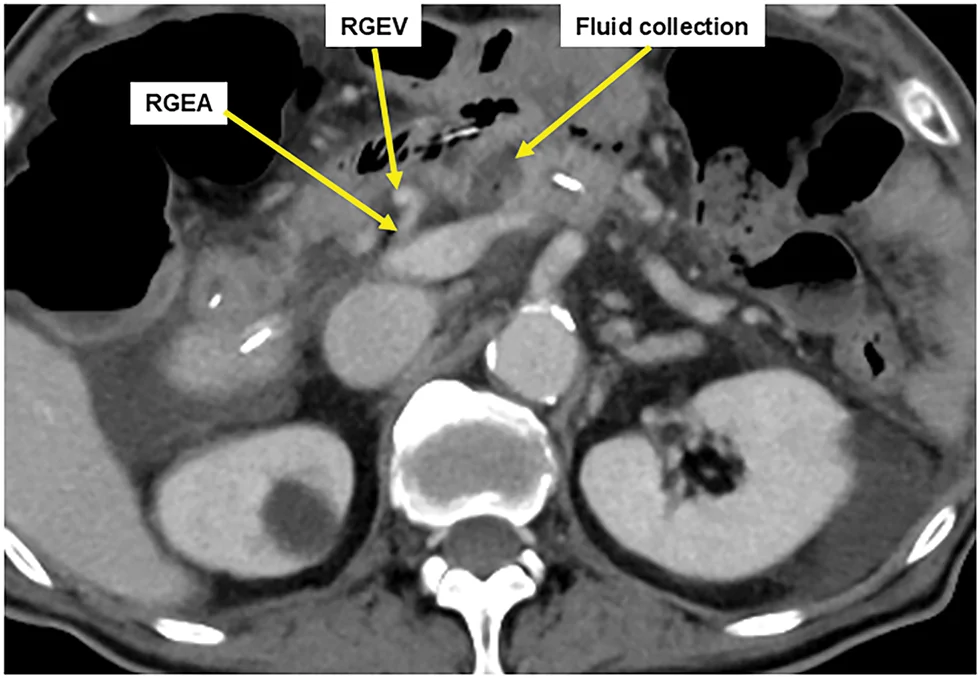
Postoperative CT findings. CT demonstrated a localized fluid collection on the cranial side of the pancreatojejunostomy, whereas only a minimal amount of fluid was observed around the preserved GDA–RGEA axis and RGEV, with no evidence of pseudoaneurysm or vascular compromise. GDA, gastroduodenal artery; RGEA, right gastroepiploic artery; RGEV, right gastroepiploic vein

PD was performed. Upon laparotomy, no unresectable factors such as peritoneal dissemination, liver metastasis, or major vascular invasion were identified. Regional lymphadenectomy, including dissection of the lymph nodes along the superior pancreatic border and within the hepatoduodenal ligament, was performed before gastric transection. During hepatoduodenal ligament dissection, a small-caliber RGA was identified intraoperatively, ligated, and divided. The right gastric vein was not clearly identified intraoperatively; therefore, it was not intentionally preserved. Gastric transection was performed immediately proximal to the pyloric ring without preserving the pyloric ring. As this step is usually executed at an early stage of PD, gastric transection was intentionally delayed until immediately before dissection of the GDA to minimize tension on the preserved RGEA and RGEV. The GDA was circumferentially skeletonized by dissection along the adventitial plane to achieve adequate oncological clearance while preserving the GDA–RGEA axis. All small branches were meticulously ligated and divided (**Fig. 2A**). Additionally, the anterior superior pancreaticoduodenal artery branching from the RGEA was ligated and divided. Pancreatic transection and resection of the pancreatic head nerve plexus were then completed, and the specimen was removed (**Fig. 2B** and **2C**). Subsequently, 12.5 mg of ICG was administered intravenously, and perfusion of the remnant stomach was assessed using the VISERA ELITE II infrared imaging system (Olympus, Tokyo, Japan). Adequate arterial perfusion was confirmed by homogeneous fluorescence of the remnant stomach without apparent perfusion defects. Adequate venous return was confirmed by the absence of congestion and the normal color of the remnant stomach. Postoperative contrast-enhanced CT also demonstrated good patency of the preserved RGEA and RGEV (**Fig. 3**). Reconstruction was performed using the modified Child method. To spatially separate the anastomosis from the preserved GDA–RGEA axis and mitigate the risk of vascular injury in the event of a postoperative pancreatic fistula, a pancreatojejunostomy was created ventral to the preserved RGEV. This allowed the vein to course dorsal to the anastomosis and lie between the anastomosis and the preserved GDA–RGEA axis (**Fig. 4**). Pancreatojejunostomy was performed using a modified Blumgart technique with placement of a lost stent. Three intra-abdominal drains were placed at the foramen of Winslow and at the cranial and caudal sides of the pancreatojejunostomy. The operative time was 530 min, with a blood loss of 1400 mL. Histopathological examination revealed pancreatic ductal adenocarcinoma, classified as pT2N0M0, Stage IB according to UICC, 8th edition, and an R0 resection was achieved (**Fig. 5**). According to the General Rules for the Study of Pancreatic Cancer (8th edition),^9)^ the pathological findings were as follows: muc > wel, Ph, TS2 (27 mm), nodular type, int, INFb, Ly1a, V1a, Pn1a, mpd0, pT3, pCH0, pDU1, pS1, pRP1, pPV0, pA0, pPL0, pOO0, pCM0, pBCM0, pDPM0, R0, pN0 (0/25), CY0, and pStage IIA. In addition, the surgical margin around the preserved GDA–RGEA axis was histopathologically negative. The postoperative course was complicated by a clinically relevant postoperative pancreatic fistula (International Study Group of Pancreatic Surgery [ISGPS] Grade B) and cholangitis, both of which improved with conservative management. On POD 7, the amylase level in the drain placed at the cranial side of the pancreatojejunostomy was 16028 U/L, and the pancreatic fistula was classified as clinically relevant (ISGPS Grade B) because prolonged drainage and drain exchange were required. The drain at the caudal side of the pancreatojejunostomy was removed on POD 5, whereas the drain placed at the foramen of Winslow was removed on POD 11. The drain placed at the cranial side of the pancreatojejunostomy was exchanged on POD 7 and 14, and was finally removed on POD 20. Intravenous antibiotics were administered from POD 4 to POD 16. Postoperative contrast-enhanced CT (**Fig. 6**) demonstrated a localized fluid collection on the cranial side of the pancreatojejunostomy, whereas only a minimal amount of fluid was observed around the preserved GDA–RGEA axis and RGEV, with no evidence of pseudoaneurysm or vascular compromise. Oral intake was well tolerated, and the patient was discharged on POD 40. Eight months after surgery, the patient remains well, with no significant change in body weight compared with the preoperative level, and is receiving S-1 adjuvant chemotherapy with no evidence of recurrence. Both CEA and CA19-9 levels normalized within several months of surgery.

## DISCUSSION

PD in patients with a history of proximal gastrectomy remains technically demanding because preservation of an adequate arterial blood supply to the remnant stomach is essential. After proximal gastrectomy, the arterial inflow to the remnant stomach is mainly maintained by the RGA and RGEA, while venous drainage occurs predominantly through the RGEV.^3)^ In standard PD, these vessels are routinely divided, which has traditionally led surgeons to perform completion total gastrectomy to avoid ischemic complications in the remnant stomach.^4)^ However, previous reports by Hirashita et al.^3)^ and Nakane et al.^6)^ demonstrated the feasibility of preserving the right gastroepiploic vessels after proximal gastrectomy.

Building on these previous reports, the present case highlights 2 technical refinements that may further improve procedural safety. First, gastric transection was intentionally delayed until immediately before dissection of the GDA to minimize tension on the preserved vessels. Second, the pancreatojejunostomy was created ventral to the RGEV while the vein was routed dorsal to the anastomosis, thereby increasing the distance between the pancreatojejunostomy and the preserved GDA–RGEA axis. To our knowledge, these technical refinements have not been specifically described in previous reports and may reduce the risk of vascular injury, particularly in the event of a postoperative pancreatic fistula.

The rationale for these 2 modifications warrants further discussion. First, gastric transection, which is typically performed at an early stage of PD, was intentionally delayed until immediately before dissection of the GDA. This approach minimizes inadvertent tension on the preserved vessels and reduces the risk of injury during lymphadenectomy and vascular dissection. However, a potential drawback is the reduced operative exposure, which can increase the technical difficulty of the procedure. Second, the reconstruction was carefully designed to protect the preserved vascular axis. When the pancreatojejunostomy is created dorsal to the RGEV, the preserved GDA–RGEA axis may course either ventral or dorsal to the anastomosis (**Fig. 4A** and **4B**); however, in both configurations, the arterial axis remains in close proximity to the pancreatojejunostomy and may therefore be vulnerable to pancreatic leakage in the event of a postoperative pancreatic fistula, potentially increasing the risk of vascular complications such as pseudoaneurysm. In contrast, in the present case, the pancreatojejunostomy was performed ventral to the RGEV, allowing the vein to course dorsal to the anastomosis (**Fig. 4C**). This configuration increased the distance between the preserved GDA–RGEA axis and the pancreatojejunostomy while positioning the RGEV between them. As a result, the RGEV may function as a physical barrier against pancreatic leakage and potentially reduce the risk of vascular complications associated with a postoperative pancreatic fistula. One potential limitation of this approach is that the remnant stomach may become relatively fixed in a cranial position. In the present case, despite the development of a clinically relevant postoperative pancreatic fistula (ISGPS Grade B), no vascular complications, such as pseudoaneurysm or hemorrhage, occurred. Although preservation of the GDA–RGEA axis was selected in the present case, preservation of a well-developed RGA may be an alternative strategy in selected patients when oncologically feasible. However, in this patient, the RGA was extremely small and was unlikely to provide sufficient perfusion to the remnant stomach.

Furthermore, although microscopic lymphatic, venous, and perineural invasion was present, histopathological examination confirmed negative surgical margins, including the margin around the preserved GDA–RGEA axis. These pathological findings suggest that preservation of the vascular pedicle did not compromise the microscopic margin status in the present case. However, this finding should not be generalized. Preservation of the GDA–RGEA axis should be considered only in carefully selected patients. When the tumor is closely adjacent to the vascular pedicle or adequate oncological dissection cannot be achieved intraoperatively, combined resection of the GDA–RGEA axis should be performed without hesitation to ensure oncological radicality.

Intraoperative assessment using ICG fluorescence imaging confirmed adequate perfusion of the remnant stomach, supporting the safety of vessel preservation in this setting.

**Fig. 7:**
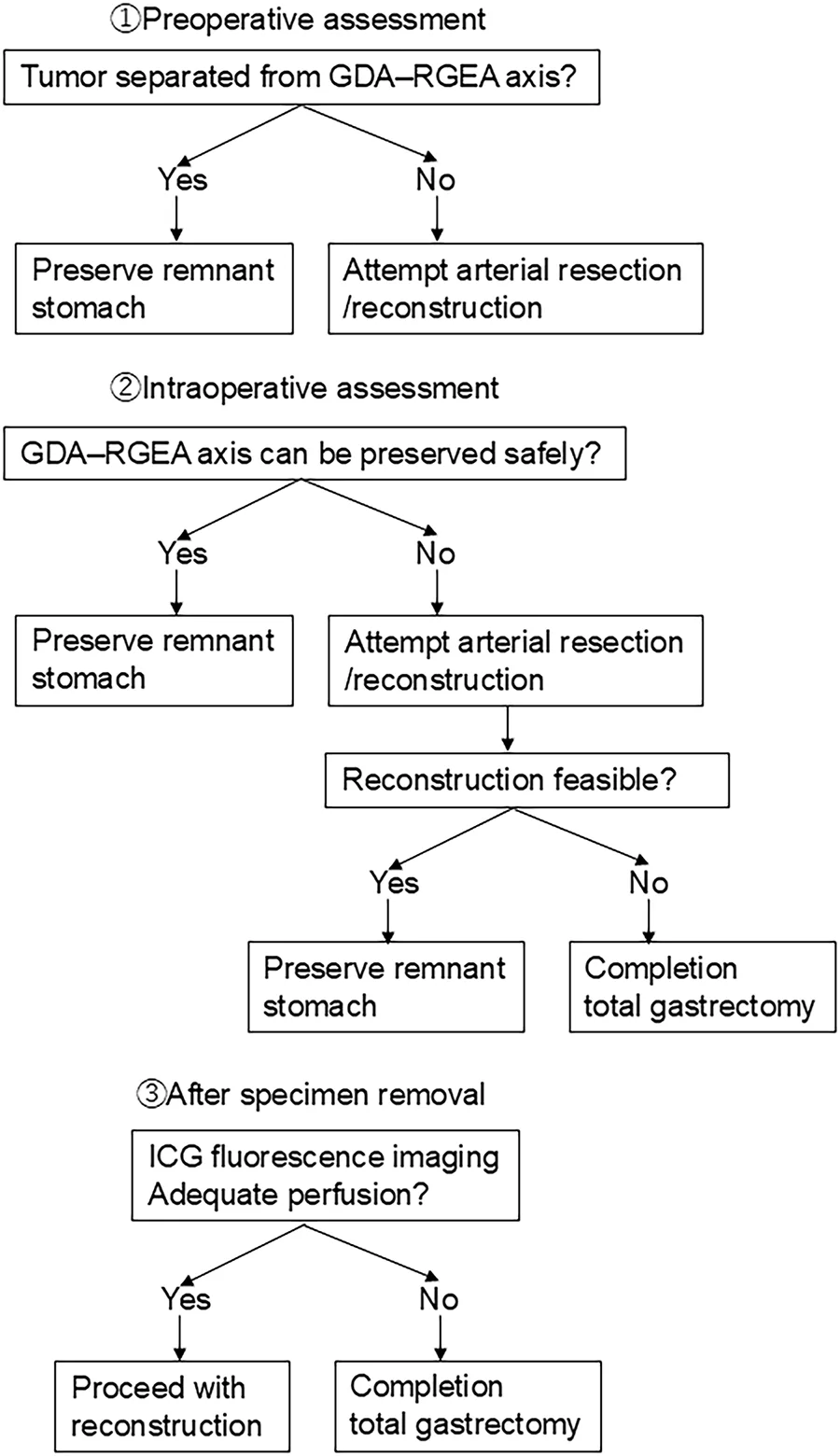
Intraoperative decision-making algorithm. Proposed intraoperative algorithm for remnant stomach preservation during PD after proximal gastrectomy. Preservation is considered when preoperative imaging demonstrates adequate separation between the pancreatic tumor and the GDA–RGEA axis. During surgery, if preservation of the GDA–RGEA axis is technically difficult or vascular injury occurs inadvertently, arterial reconstruction should be attempted. If reconstruction is not feasible, conversion to completion total gastrectomy is recommended. After specimen removal, remnant gastric perfusion should be assessed using ICG fluorescence imaging. If adequate perfusion cannot be confirmed, a completion total gastrectomy should be considered. GDA, gastroduodenal artery; ICG, indocyanine green; PD, pancreaticoduodenectomy; RGEA, right gastroepiploic artery

Based on our experience, we propose a simple intraoperative decision-making algorithm for remnant stomach preservation during PD after proximal gastrectomy (**Fig. 7**). Careful preoperative assessment of the relationship between the tumor and the GDA–RGEA axis is the first prerequisite. During surgery, preservation of the remnant stomach should be attempted only when adequate oncological clearance and safe vascular preservation can be achieved. If preservation of the GDA–RGEA axis is not technically feasible or the vessels are inadvertently injured, arterial reconstruction should be considered first. When satisfactory reconstruction cannot be achieved, conversion to completion total gastrectomy should be performed. Finally, remnant gastric perfusion should be confirmed using ICG fluorescence imaging before reconstruction. If adequate perfusion cannot be confirmed, a completion total gastrectomy should be considered. Although evidence regarding reconstruction of the RGEA is limited, arterial resection and reconstruction of a replaced right hepatic artery during PD has been reported to provide acceptable oncological outcomes in carefully selected patients.^10)^ These reports support consideration of vascular reconstruction before completion total gastrectomy when oncological radicality can be maintained.

In addition to technical feasibility, perioperative systemic chemotherapy plays a crucial role in improving the outcomes of pancreatic ductal adenocarcinoma.^11)^ In the present case, preservation of the remnant stomach may have contributed to maintaining postoperative oral intake, as no substantial postoperative weight loss was observed; the patient’s body weight was 47.6 kg before surgery and 48.0 kg at 3 months postoperatively. This consideration was particularly important because of the patient’s advanced age. Proximal gastrectomy provides better preservation of postoperative body weight and nutritional status than total gastrectomy, particularly in older patients.^12)^ Because PD itself can adversely affect postoperative nutritional status,^13)^ additional completion total gastrectomy might have further impaired postoperative oral intake and nutritional recovery in this patient. Although the patient’s body weight remained stable, the prognostic nutritional index decreased from 45.0 to 38.7 during the same period. Therefore, any potential nutritional benefit of remnant stomach preservation should be interpreted cautiously. Nevertheless, preservation of the remnant stomach may have facilitated the continuation of adjuvant chemotherapy in this patient.

## CONCLUSIONS

PD with preservation of the RGEA and RGEV after proximal gastrectomy is feasible in carefully selected patients. Delaying gastric transection and positioning the RGEV between the pancreatojejunostomy and the preserved GDA–RGEA axis are important technical considerations for protecting the preserved vessels. This approach may allow oncologically appropriate resection while maintaining remnant gastric perfusion and postoperative gastric function in carefully selected patients.

## DECLARATIONS

### Availability of data and materials

The data are not available for public access because of patient privacy concerns but are available from the corresponding author upon reasonable request.
